# An aqueous *Commiphora myrrha* extract ameliorates paclitaxel-induced peripheral neuropathic pain in mice

**DOI:** 10.3389/fphar.2023.1295096

**Published:** 2023-12-21

**Authors:** Altaf Al-Romaiyan, Ahmad Barakat, Liny Jose, Willias Masocha

**Affiliations:** Department of Pharmacology and Therapeutics, College of Pharmacy, Kuwait University, Kuwait

**Keywords:** paclitaxel, peripheral neuropathy, plant extract, thermal hyperalgesia, mechanical allodynia

## Abstract

**Background:** Chemotherapy-induced neuropathic pain (CINP) is a debilitating side effect in individuals undergoing cancer treatment. Treatment of CINP with the current available classes of drugs is limited and often yields unsatisfactory results. Finding therapeutic alternatives of plant origin could provide a new way for the management of CINP. *Commiphora myrrha* (CM) resin extract has been reported to have anti-inflammatory and analgesic activities, but the effect of CM on neuropathic pain is yet to be investigated in CINP.

**Objectives:** The aim of this study was to investigate the antinociceptive effect of CM extract in a mouse model of paclitaxel-induced neuropathic pain (PINP).

**Methods:** The effects of CM on thermal hyperalgesia and mechanical allodynia were assessed in female BALB/c mice with PINP using a hot plate and a plantar aesthesiometer, respectively. Motor coordination was evaluated using a rotarod apparatus. The involvement of transient receptor potential vanilloid channel 1 (TRPV1) in CM actions was investigated using a capsaicin (a TRPV1 agonist)-induced nociception test. The genetic expression of *Trpv1*, *Nrf2*, *Sod2*, and *Hmox1* was assessed using real-time PCR, while protein expression of TRPV1, Iba-1, and CD11b was assessed using Wes™.

**Results:** Administration of CM to mice with established PINP produced a dose-dependent reduction in thermal hyperalgesia. Prophylactic treatment of mice with CM prevented the development of paclitaxel-induced thermal hyperalgesia and mechanical allodynia. CM did not change the motor coordination of mice, as the reaction latency and the rotational velocity of animals pretreated with CM extract were similar to those of animals pretreated with vehicle. CM significantly decreased the number and duration of the flick responses following capsaicin injection into the dorsal surface of the hind paw of mice. The protein expression of TRPV1 was upregulated in the spinal cord of paclitaxel-treated animals compared to vehicle-only-treated control animals, while CM-treated animals had values similar to vehicle-only-treated control animals. The mRNA expression of *Nrf2*, a major antioxidant transcription factor, was upregulated in the paw skin of mice treated with CM compared to those treated with paclitaxel alone.

**Conclusion:** These results indicate that CM may both treat established and prevent the development of paclitaxel-induced thermal hyperalgesia and mechanical allodynia without any impairment in the motor activity of mice. CM may mediate its action through the peripheral inhibition of TRPV1 channel activity, restoration of normal TRPV1 protein expression in the spinal cord, and elevation of cellular antioxidant defenses. CM has the potential to be used as a therapeutic alternative to treat CINP.

## 1 Introduction

Peripheral neuropathy is associated with neuropathic pain and loss of sensation and is most commonly caused by chronic diseases, such as diabetes, or drugs, such as chemotherapeutic drugs and some antiretroviral drugs (Marchettini et al., 2006). Chemotherapy-induced peripheral neuropathy (CIPN) is a dose-dependent adverse effect of many chemotherapeutic agents, including platinum-based antineoplastics (particularly oxaliplatin and cisplatin), the vinca alkaloids (particularly vincristine and vinblastine), the epothilones (ixabepilone), and the taxanes (paclitaxel and docetaxel) (Addington and Freimer, 2016). Approximately 30%–40% of patients receiving chemotherapy develop CIPN, which can result in harmful dose reductions and treatment cessation, and this can severely affect the quality of life of cancer survivors (Boyette-Davis et al., 2015; Addington and Freimer, 2016; Hou et al., 2018). The treatment of painful CIPN, also referred to as chemotherapy-induced neuropathic pain (CINP), may include anticonvulsants and antidepressants. However, these agents do not provide a satisfactory level of relief for many patients. A recent systemic review regarding the effectiveness of the current medications for the treatment of CINP showed either no benefit or at most moderate benefit in patients with CINP when treated with either anticonvulsants or antidepressants (Hou et al., 2018). Therefore, further research is required to identify additional preventative or curative approaches.

An active research area is the identification of new agents of plant origin for the management of pain. *Commiphora myrrha* (CM) is a plant belonging to the family Burseraceae and is native to the Arabian Peninsula (Oman and Yemen) and Africa (Somalia, Eritrea, and eastern Ethiopia) (Tucker, 1986). The gum extract of CM has been shown to possess multiple therapeutic activities. It has been reported that CM extract may have anti-inflammatory and analgesic activities (Dolara et al., 1996; Cheng et al., 2011; Su et al., 2011; Su et al., 2012; Shalaby and Hammouda, 2014; Germano et al., 2017). The chronic administration of a mixture of plant extracts containing CM extract resulted in the alleviation of thermal hypersensitivity and mechanical allodynia in rodents with chronic constriction injury of the sciatic nerve, an animal model of neuropathic pain (Hu et al., 2017; Fotio et al., 2019), through central modulation of transient receptor potential vanilloid channel 1 (TRPV1) expression (Hu et al., 2017). TRPV1, a nonselective cation channel, has been shown to play a crucial role in neuropathic pain (Boyette-Davis et al., 2015). These channels are activated by noxious heat, acidic environments, and capsaicin (White et al., 2011). It has been shown that the activation of TRPV1 resulted in pain sensitization. Indeed, the expression of TRPV1 in rat dorsal root ganglia neurons was upregulated following paclitaxel injection (Hara et al., 2013). Furthermore, using the antagonist of TRPV1 attenuated thermal hyperalgesia in a mouse model of paclitaxel-induced neuropathic pain (PINP) (Chen et al., 2011).

Oxidative stress has also been involved in the development of CIPN. Treating rodents with paclitaxel has been associated with the reduction in the expression of key antioxidant transcription factors such as nuclear factor erythroid-2-related factor 2 (NRF2), which is responsible for the expression of many antioxidant molecules including superoxide dismutase 2 (SOD2) and heme oxygenase (HO-1) (Zhao et al., 2021). Reversing this imbalance could improve CIPN and thus prevent or treat the condition.

In this study, we evaluated the ability of CM extract to prevent the development of and/or treat established paclitaxel-induced thermal hyperalgesia and mechanical allodynia in mice and investigated the possible mechanism of action underlying the antinociceptive effect of CM extract.

## 2 Materials and methods

### 2.1 Plant material

The plant name was checked with http://www.theplantlist.org. *Commiphora myrrha* (Nees) Engl. is the accepted name of a species in the genus *Commiphora* (family Burseraceae). *Commiphora myrrha* (CM) resin was used in this study and was purchased from a local market. The identification and deposition of a voucher specimen were done as previously prescribed (Al-Romaiyan et al., 2020). The preparation of the crude extract of CM was done as previously prescribed (Al-Romaiyan et al., 2020; Al-Romaiyan et al., 2023), diluted in distilled water to the concentrations detailed in the figure legends for use in the experiments, and administered to animals by oral gavage.

### 2.2 Animals

Female BALB/c mice (8–12 weeks old; 20–30 g; n = 230) were used in this study. In the current study, female mice were used following the recommendation of the members of the Sex, Gender, and Pain Special Interest Group of the International Association for the Study of Pain (IASP) that “all pain researchers consider testing their hypotheses in both sexes, or if restricted by practical considerations, only in females” (Greenspan et al., 2007). The use of the current strain of animals was chosen based on availability and our previous studies with the same strain (Parvathy and Masocha, 2012; Masocha and Thomas, 2019; Thomas et al., 2020; Al-Romaiyan and Masocha, 2022).

The animals were supplied by the Animal Resources Center at the Health Sciences Center, Kuwait University, Kuwait. The animals were handled in accordance with NIH Animal Research Advisory Committee Guidelines for the Care and Use of Laboratory Animals. All procedures were approved by the Health Sciences Center Ethical Committee for the use of Laboratory Animals in Teaching and in Research, Kuwait University (Ref: 3986/VDR/EC/, Date 03/01/2022). All animals were maintained in temperature-controlled (24°C ± 1°C), light–dark cycle (lights on at 06:00 a.m. and lights off at 06:00 p.m.) rooms and provided with food and water *ad libitum*. All experiments were performed between 0800 and 1600 h to reduce the effects of circadian variations in pharmacological effects.

### 2.3 Development of the animal model

Paclitaxel (Tocris, Bristol, United Kingdom) stock (6 mg/mL) was prepared by dissolving paclitaxel in a solution comprised 50% Cremophor EL and 50% absolute ethanol and stored at −20°C for a maximum of 14 days. On the day of the experiment, paclitaxel stock was diluted to 0.2 mg/mL in normal saline (NaCl 0.9%) just before injection. The vehicle for paclitaxel which comprised a mixture of Cremophor EL and ethanol was diluted at the time of injection with normal saline in the same proportion as the paclitaxel solution. Paclitaxel (2 mg/kg) or its vehicle was intraperitoneally (i.p.) injected into mice at a dose of 10 μL/g body mass, once per day for 5 consecutive days.

### 2.4 Drug administration protocol

**FIGURE 1:**
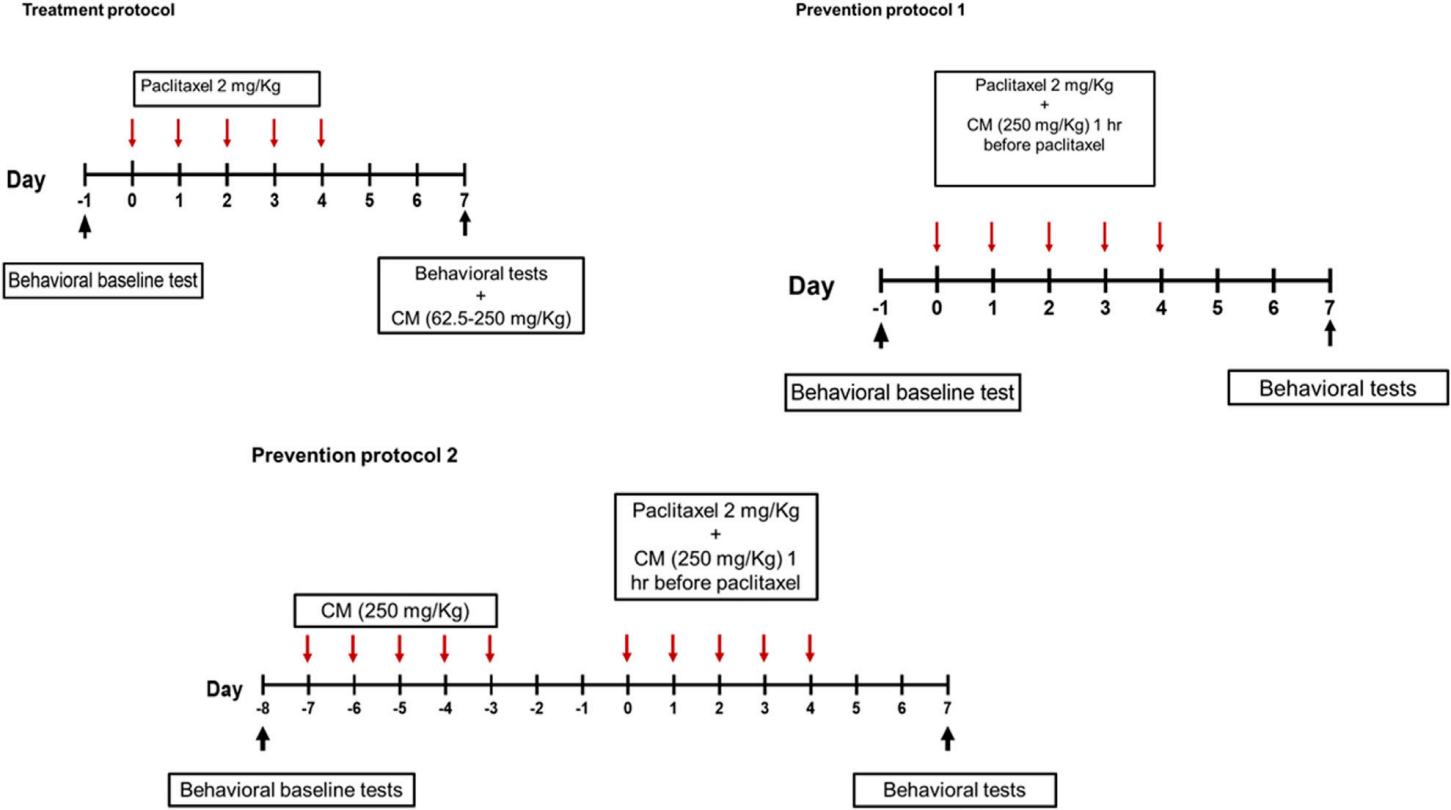
Schematic representation of paclitaxel and drug administration protocols.

To evaluate the antihyperalgesic and antiallodynic effects of CM, CM was administered by oral gavage following two main different protocols (Figure 1): treatment and prevention protocols. The plant extract or its vehicle was administered to paclitaxel-treated animals. A control group with no treatment was always included in all protocols. All behavioral studies were not blinded but were carried out by three different researchers independent of each other.

### 2.5 Thermal nociception

Thermal hyperalgesia in mice was measured using the hot plate (Panlab SL, Barcelona, Spain) as previously described (Masocha, 2014). Reaction latencies to the hot plate test were assessed both before (baseline latency) and on day 7 after the initial injection of paclitaxel. In brief, mice were individually positioned on a hot plate with the temperature adjusted to 55°C ± 1°C. The time to the first sign of nociception such as paw licking, flinching, or jump response to avoid the heat was recorded, and the animals were immediately removed from the hot plate. To avoid damage to the paws, a cut-off period of 20 s was maintained.

### 2.6 Mechanical allodynia

Mechanical allodynia in mice was measured using the dynamic plantar aesthesiometer (Ugo Basile, Gemonio, Italy) as previously described (Al-Romaiyan and Masocha, 2022) and following the procedures described by the manufacturer. In brief, mice were placed inside plastic chambers on the top of a perforated metal platform for approximately an hour to habituate. Under the perforated platform, a movable touch-stimulator unit with a 0.5-mm metal filament was placed. This unit had been calibrated according to the manufacturer’s instructions. Once the start key on the microprocessor-controlled electronic unit was pressed, the automated 0.5-mm metal filament was lifted onto the plantar of the hind paw, and a force that was linearly increased (0.25 g/s) was applied to the hind paw. Once the mouse withdrew its paw, the dynamic plantar aesthesiometer automatically recognized and recorded the force at the time of paw withdrawal. A cut-off force of 5 g was maintained. The hind paws of each mouse were tested at least four times with a 2-min interval between each reading.

### 2.7 Capsaicin test

The effect of CM on capsaicin-induced nociception was evaluated as described previously (Sałat and Filipek, 2015). Capsaicin is a selective TRPV1 agonist. In brief, the mice underwent an adaptation period of 15 min, following which capsaicin was injected into the dorsal surface of the right hind paw of a mouse. Capsaicin was prepared by dissolving 5 μg of capsaicin in 20 μL of physiological saline and ethanol (5:1, v/v). CM (250 mg/kg) or its vehicle (water) was given orally to the mouse 1 h and 30 min before capsaicin administration. The mice were observed for 5 min after capsaicin injection. Reaction to pain such as the number and amount of time spent on licking, biting, flinching, or lifting the injected paw was measured using a counter and a chronometer.

### 2.8 Motor coordination

The motor coordination of the mice was evaluated using the rotarod apparatus (Panlab SL, Barcelona, Spain). An accelerating mode from 4 to 40 rotations per minute (rpm) over 5 min was used as described previously (Galante et al., 2009). All mice were trained for 3 days. On the day of the experiment, mice received 250 mg/kg CM or its vehicle (water) orally before the test. The latency (in seconds) and the rotational velocity (in rpm) for the first fall were recorded 1 h and 30 min after the administration of CM or its vehicle. The cut-off time was set at 300 s.

### 2.9 RNA extraction and real-time PCR

Tissues (brain, spinal cord, and paw skin) from animals were dissected and snap-frozen in liquid nitrogen before storing at −80°C. RNA was extracted using the QIAGEN RNeasy kit, and cDNA was synthesized using SuperScript™ Reverse Transcriptase (Invitrogen, United States) as previously prescribed (Masocha, 2009; Al-Romaiyan and Masocha, 2022). Real-time PCR was performed using a QuantStudio™ 7 Flex Real-Time PCR System (Applied Biosystems) as described previously (Masocha, 2009; Al-Romaiyan and Masocha, 2022). The primers for *Trpv1*, *Nrf2*, *Sod2*, *Hmox1*, and *Ppia* (cyclophilin), as shown in Table 1, were purchased from Sigma-Aldrich (United States). The threshold cycle (Ct) values for all cDNA samples were obtained, and the mRNA levels for each sample were normalized to *Ppia* (a housekeeping gene) whose expression was presented as ΔCt. The 2^−ΔΔCt^ method was used to calculate the relative expression of the gene of interest (Livak and Schmittgen, 2001).

**TABLE 1:** Sequences of primers used in real-time RT-PCR.

Gene name	Polarity	Primer sequence (sense and anti-sense) (5′ → 3′)	Annealing temperature (°C)
*Nuclear factor erythroid*-*2*-*related factor 2* (*Nrf2*)	Sense	CAG​CAT​AGA​GCA​GGA​CAT​GGA​G	60°C
	Anti-sense	GAA​CAG​CGG​TAG​TAT​CAG​CCA​G	
*Superoxide dismutase 2*, mitochondrial (*Sod2*)	Sense	TAA​CGC​GCA​GAT​CAT​GCA​GCT​G	60°C
	Anti-sense	AGG​CTG​AAG​AGC​GAC​CTG​AGT​T	
*Heme oxygenase 1* (*Hmox*-*1*)	Sense	CAC​TCT​GGA​GAT​GAC​ACC​TGA​G	60°C
	Anti-sense	GTG​TTC​CTC​TGT​CAG​CAT​CAC​C	
*Transient receptor potential cation channel subfamily V member 1* (*Trpv1*)	Sense	ACC​ACG​GCT​GCT​TAC​TAT​CG	60°C
	Anti-sense	TCC​CCA​ACG​GTG​TTA​TTC​AG	
*Cyclophilin* (*Ppia*)	Sense	GCTTTTCGCCGCTTGCT	60°C
	Anti-sense	CTCGTCATCGGCCGTGAT	

### 2.10 Wes™ capillary-based protein electrophoresis

Tissues were homogenized with RIPA lysis buffer (Millipore, United States) containing protease and phosphatase inhibitors (Sigma-Aldrich, United States). The samples were centrifuged at 4°C in a microcentrifuge at 13,000 rpm for 15 min, and the supernatant was transferred to a clean tube on ice. Protein concentrations were measured following the manufacturer’s protocol using the Pierce BCA kit (Invitrogen, United States). Proteins were separated and quantified by Wes™ capillary-based protein electrophoresis as previously described (Aly et al., 2020; Al-Romaiyan et al., 2023), using the 12–230 kDa separation module (ProteinSimple), and analyzed using Compass for Simple Western software (ProteinSimple). The primary antibodies for TRPV1 (Invitrogen PA1-29421), Iba-1 (Boster Biological Technologies M01394), CD11b (Abcam 133357), and anti-β-actin (Cell Signaling Technology, United States) were diluted using an antibody diluent (anti-β-actin 1:50, TRPV1 1:10, Iba-1 1:50, and CD11b 1:50), following the manufacturer’s recommendations. The ratio of the area of the electropherogram for the protein of interest to the area of the electropherogram for the housekeeping protein (β-actin) was calculated and normalized to the control group.

### 2.11 Statistical analysis

Data are represented as mean ± SEM for normally distributed data or median and interquartile range for skewed data. Differences between treatment groups were assessed using unpaired Student’s *t*-test or one-way or two-way analysis of variance (ANOVA) and Bonferroni’s multiple comparison test or Dunn’s multiple comparison test if data are normally distributed and Kruskal–Wallis and Dunn’s multiple comparison tests if data are not normally distributed. All statistical analyses were performed using GraphPad Prism software (version 10.0), and differences were considered significant at *p* < 0.05.

## 3 Results

### 3.1 Effect of acute CM extract administration on paclitaxel-induced thermal hyperalgesia

**FIGURE 2:**
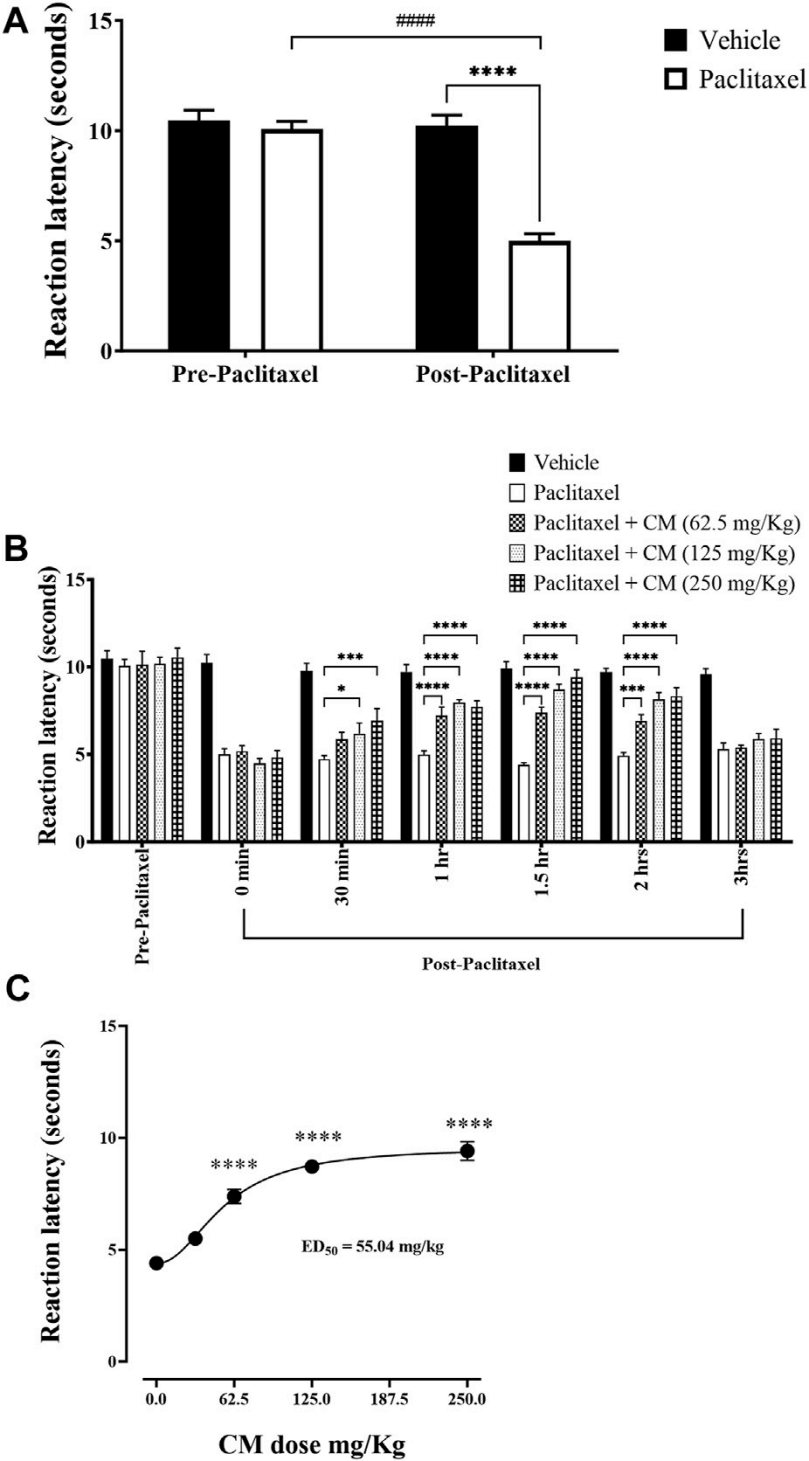
Antihyperalgesic effects of acute oral CM extract administration in female BALB/c mice with paclitaxel-induced thermal hyperalgesia at day 7 post-paclitaxel injection. **(A)** Reaction latencies to heat were measured in paclitaxel-treated female BALB/c mice compared to vehicle-treated counterparts. **(B)** Reaction latencies to heat were measured before (0 min) and after (30, 60, 90, 120, and 180 min) oral CM extract (62.5–250 mg/kg) administration. Each bar represents the mean + SEM of values obtained from 9–13 animals per treatment group. *p < 0.05, ***p < 0.001, and ****p < 0.0001 compared to the paclitaxel group post-paclitaxel administration and ####*p* < 0.0001 compared to the paclitaxel group at baseline (two-way ANOVA followed by Bonferroni’s multiple comparison *post hoc* tests). **(C)** Dose–response curve of reaction latencies to heat at 1.5 h after CM (31.25–250 mg/kg) administration. Data are mean + SEM of values obtained from 4–13 animals per treatment group. ****p < 0.0001 compared to the paclitaxel group (one-way ANOVA followed by Dunn’s multiple comparison *post hoc* tests).

Injection of paclitaxel caused a significant reduction in reaction latency to thermal (hot) nociception at day 7 post-paclitaxel injection. Paclitaxel-treated mice had reduced reaction latency on day 7 compared to the baseline latency and vehicle-treated animals (*p* < 0.0001), whereas there was no significant change in vehicle-treated mice on day 7 compared to baseline latency (*p* > 0.05; Figure 2A).

Acute administration of CM extract (31.25–250 mg/kg) to female BALB/c mice that had developed paclitaxel-induced thermal hyperalgesia resulted in an increase in reaction latencies at all specified time points. The antihyperalgesic effect of CM was noticeable as early as 30 min. The maximum antihyperalgesic effect of CM extract of all concentrations used was achieved at 1.5 h after CM extract administration and at a CM dose of 250 mg/kg, which completely restored the reaction latency to vehicle-treated control values. However, this antihyperalgesic effect of CM was attenuated after 3 h of CM administration (Figure 2B). The antihyperalgesic effect of CM was dose-dependent (Figure 2C). Based on this dose–activity profile of CM on thermal hyperalgesia, CM at a dose of 250 mg/kg was used in all subsequent experiments.

### 3.2 Effect of chronic CM extract administration on paclitaxel-induced thermal hyperalgesia

**FIGURE 3:**
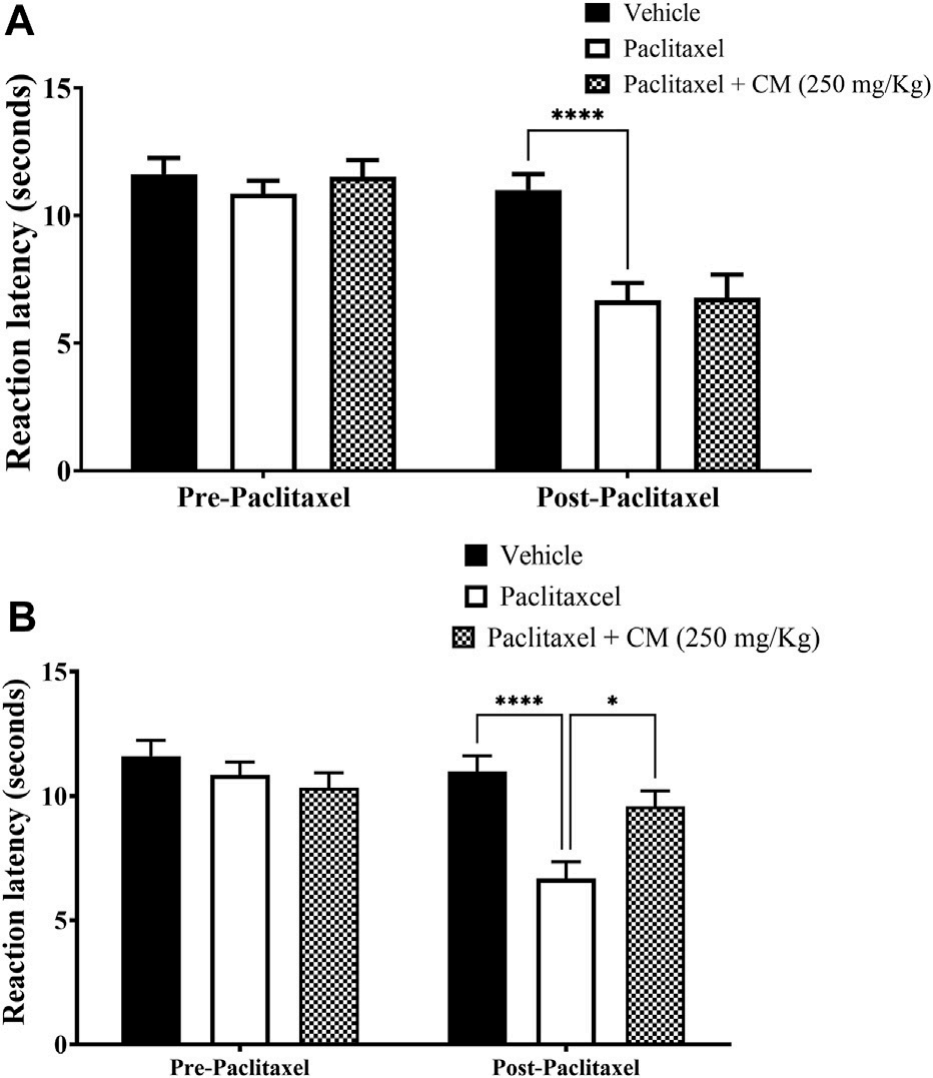
Chronic oral CM extract administration in female BALB/c mice prevents the development of paclitaxel-induced thermal hyperalgesia at day 7 post-paclitaxel injection. **(A)** CM extract (250 mg/kg) was administered to female BALB/c mice in conjunction with paclitaxel injection for 5 days. Reaction latencies to heat were measured before and after oral CM administration. Each bar represents the mean + SEM of values obtained from five animals. **(B)** CM extract (250 mg/kg) administered to female BALB/c mice for 10 days (5 days before paclitaxel injection and 5 days during paclitaxel injection). Reaction latencies to heat were measured before and after oral CM administration. Each bar represents the mean + SEM of values obtained from 8–13 animals per treatment group. *p < 0.05 and ****p < 0.0001 compared to the paclitaxel group post-paclitaxel (two-way ANOVA followed by Bonferroni’s multiple comparison *post hoc* tests).

CM extract at 250 mg/kg did not prevent the development of paclitaxel-induced thermal hyperalgesia when co-administered with paclitaxel for 5 days (prevention protocol 1; Figure 3A). However, when the mice were pretreated with CM (250 mg/kg) for 5 days before and then treated with CM for another 5 days during paclitaxel injection (total CM treatment time is 10 days), CM prevented the development of paclitaxel-induced thermal hyperalgesia (prevention protocol 2; Figure 3B).

### 3.3 Effect of chronic CM extract administration on paclitaxel-induced mechanical allodynia

**FIGURE 4:**
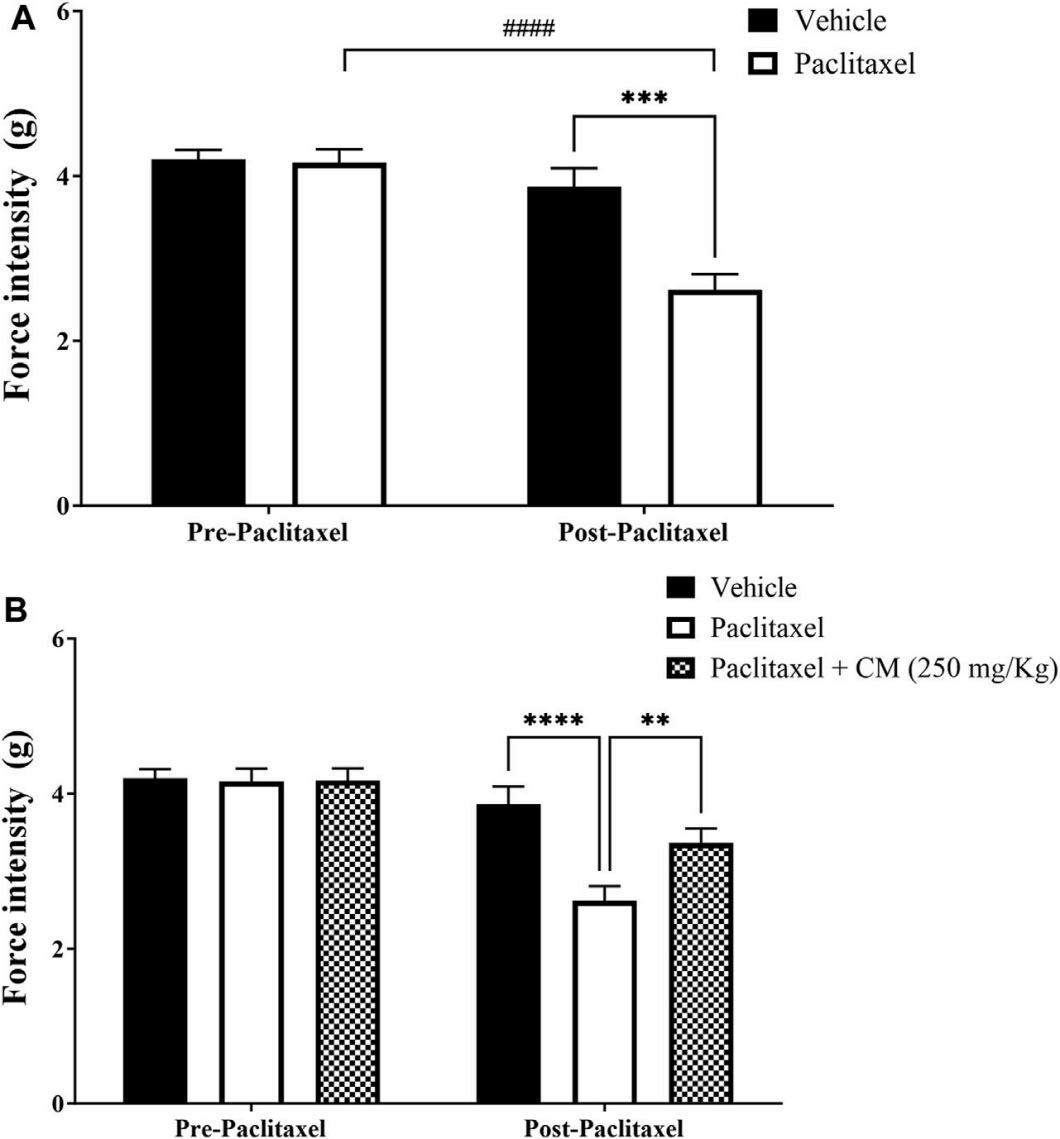
Chronic oral CM extract administration in female BALB/c mice prevents the development of paclitaxel-induced mechanical allodynia at day 7 post-paclitaxel injection. **(A)** Withdrawal thresholds to the mechanical stimulus were measured in paclitaxel-treated female BALB/c mice compared to vehicle-treated counterparts. **(B)** CM extract (250 mg/kg) administered to female BALB/c mice for 10 days (5 days before paclitaxel injection and 5 days during paclitaxel injection). Withdrawal thresholds to the mechanical stimulus were measured before and after oral CM administration. Each bar represents the mean + SEM of values obtained from 9–10 animals per treatment group. **p < 0.01, ***p < 0.001, and ****p < 0.0001 compared to the paclitaxel group post-paclitaxel and ####*p* < 0.0001 compared to the paclitaxel group at baseline (two-way ANOVA followed by Bonferroni’s multiple comparison *post hoc* tests or Dunn’s multiple comparison *post hoc* tests).

Paclitaxel treatment induced mechanical allodynia in mice on day 7 after treatment. There was a reduction in the withdrawal threshold to mechanical stimuli on day 7 compared to the baseline withdrawal threshold before treatment with paclitaxel and vehicle-treated animals on day 7 (*p* < 0.0001 vs. baseline, *p* < 0.001 vs. vehicle-treated animals), whereas there was no significant change in vehicle-treated mice on day 7 compared to baseline latency (*p* > 0.05; Figure 4A).

Pretreating BALB/c mice for 5 days before and 5 days during paclitaxel injection (prevention protocol 2) with CM (250 mg/kg) ameliorated paclitaxel-induced mechanical allodynia (prevention protocol 2; Figure 4B).

### 3.4 Effect of CM extract administration on capsaicin-induced nociception

**FIGURE 5:**
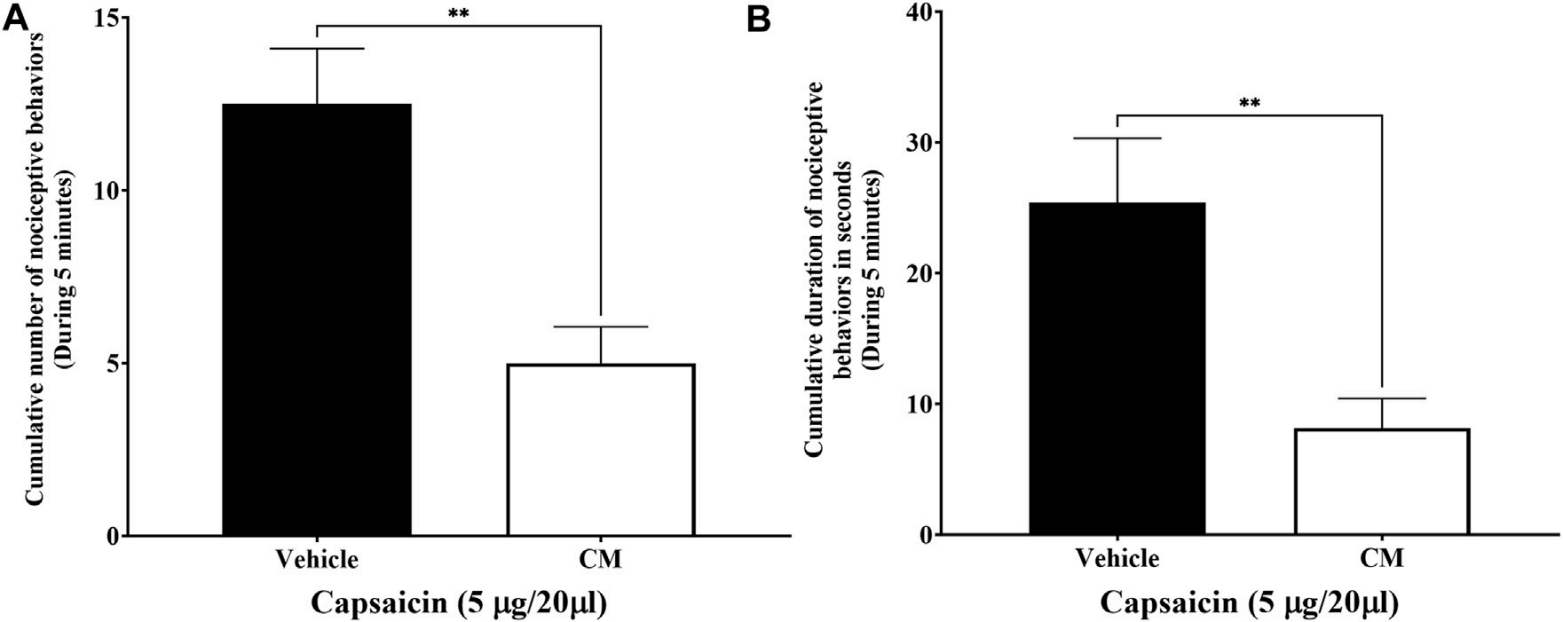
Oral CM extract ameliorates capsaicin-induced nociception in female BALB/c mice. CM extract (250 mg/kg) or its vehicle was administered orally to female BALB/c mice 1 h and 30 min before injecting capsaicin (5 µg/20 µL) into the dorsal surface of the right hind of the mice. Nociceptive behaviors in response to capsaicin such as the number **(A)** and the amount of time **(B)** spent on licking, biting, flinching, or lifting the injected paw were measured over 5 min. Each bar represents the mean + SEM of values obtained from eight animals per treatment group. **p < 0.01 compared to the vehicle-treated group (unpaired Student’s *t*-test).

Injection of capsaicin, a selective TRPV1 agonist, into the dorsal surface of the right hind paw of the mice caused the mice to lick, bite, flinch, or lift the injected paw several times (Figure 5A) and for a duration of approximately 25.4 ± 4.927 s (Figure 5B), while the mice injected with vehicle for capsaicin did not produce any of those responses. These responses were significantly reduced when mice were pretreated with CM extract (250 mg/kg) 1.5 h before capsaicin administration compared to mice pretreated with the vehicle for CM. Both the number and duration of the capsaicin-induced nociceptive responses were decreased in CM-treated mice (*p* < 0.01; Figure 5).

### 3.5 Effect of CM extract administration on motor coordination

**FIGURE 6:**
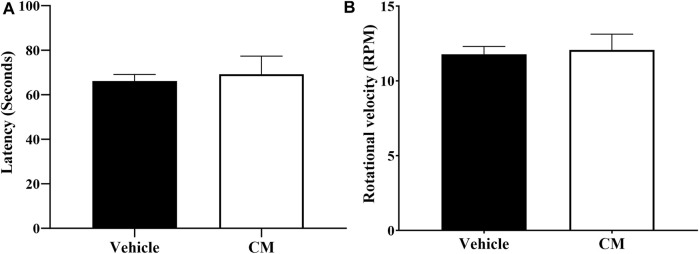
Effect of oral CM extract administration on motor coordination in female BALB/c mice. CM extract (250 mg/kg) or its vehicle was administered orally to female BALB/c mice, and latency in seconds **(A)** and rotational velocity in rpm **(B)** for the first fall was recorded 1 h and 30 min after administration of CM or its vehicle using the rotarod apparatus. Each bar represents the mean + SEM of values obtained from nine animals per treatment group.

Administration of 250 mg/kg CM extract to mice did not affect their motor coordination (Figure 6) as measured using the rotarod apparatus. The latency (Figure 6A) and the rotational velocity (Figure 6B) of mice pretreated with CM extract were similar to those of mice pretreated with vehicle.

### 3.6 Effect of CM extract administration on mRNA expression of key antioxidative stress mediators and mRNA and protein expression of TRPV1

**FIGURE 7:**
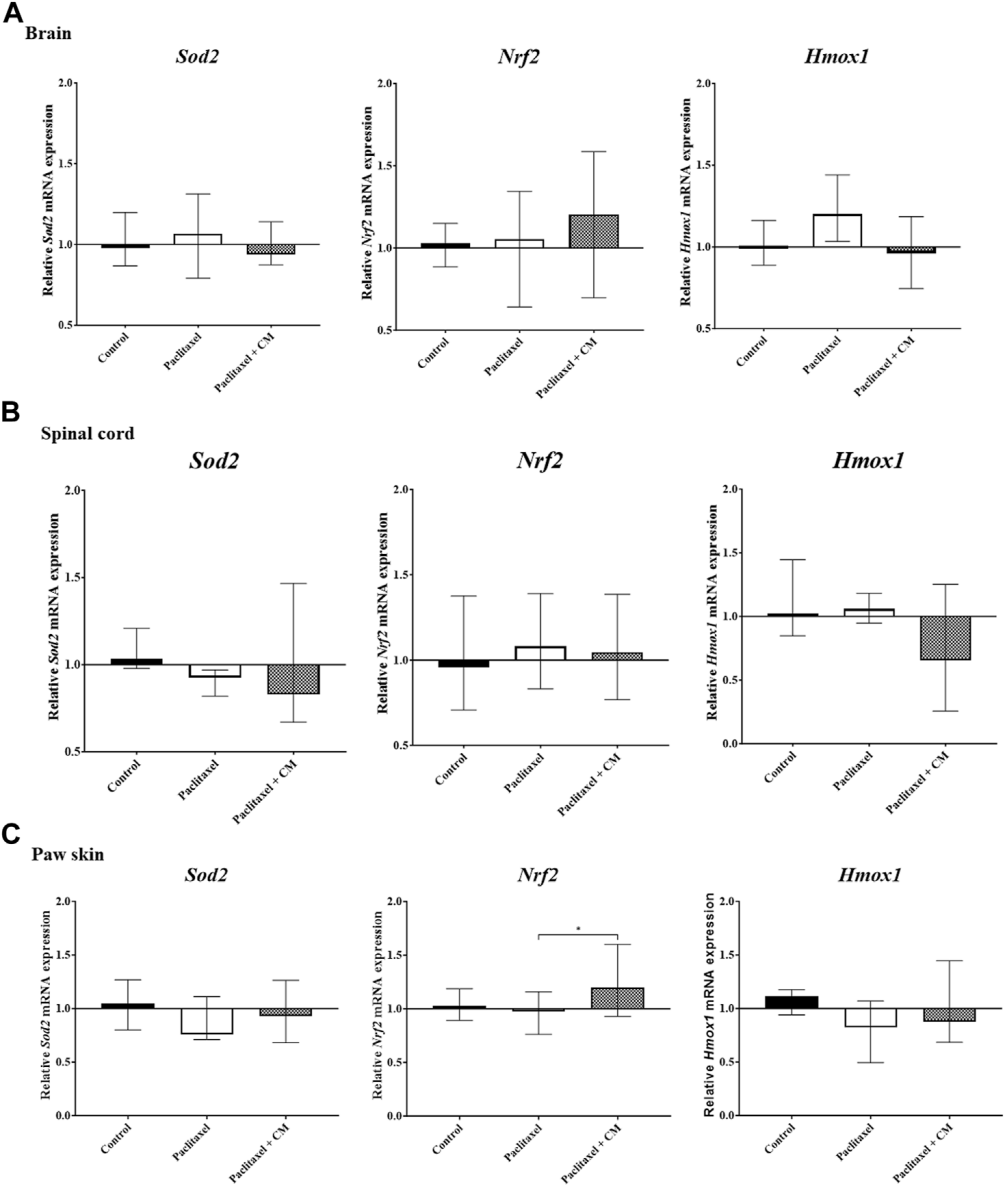
Effect of oral CM extract administration on the relative mRNA expression of Sod2, Nrf2, and Hmox1 in mice treated with paclitaxel. CM extract (250 mg/kg) was administered to female BALB/c mice for 10 days (5 days before paclitaxel injection and 5 days during paclitaxel injection). At day 7 post-paclitaxel injection, tissues were dissected, and the relative expression of *Sod2*, *Nrf2*, and *Hmox1 mRNA* in the brain **(A)**, spinal cord **(B)**, and paw skin **(C)** of the mice was measured. Each bar represents the median and interquartile range of values obtained from 6–7 animals per treatment group (for brain and spinal cord analysis) and 15 animals per treatment group (for paw skin analysis). *p < 0.05 compared to the paclitaxel-treated group (Kruskal–Wallis test followed by Dunn’s multiple comparison *post hoc* test).

The mRNA expression of Nrf2, Sod2, and Hmox1 in the tissues extracted from the brain and spinal cord of paclitaxel + CM-treated animals (prevention protocol 2) did not change compared to paclitaxel + vehicle- or vehicle-only-treated animals (Figures 7A, B). However, paclitaxel + CM-treated animals had a significant increase in the mRNA expression of Nrf2 but not Sod2 and Hmox1 in the paw skin (Figure 7C) when compared to paclitaxel + vehicle- or vehicle-only-treated animals.

**FIGURE 8:**
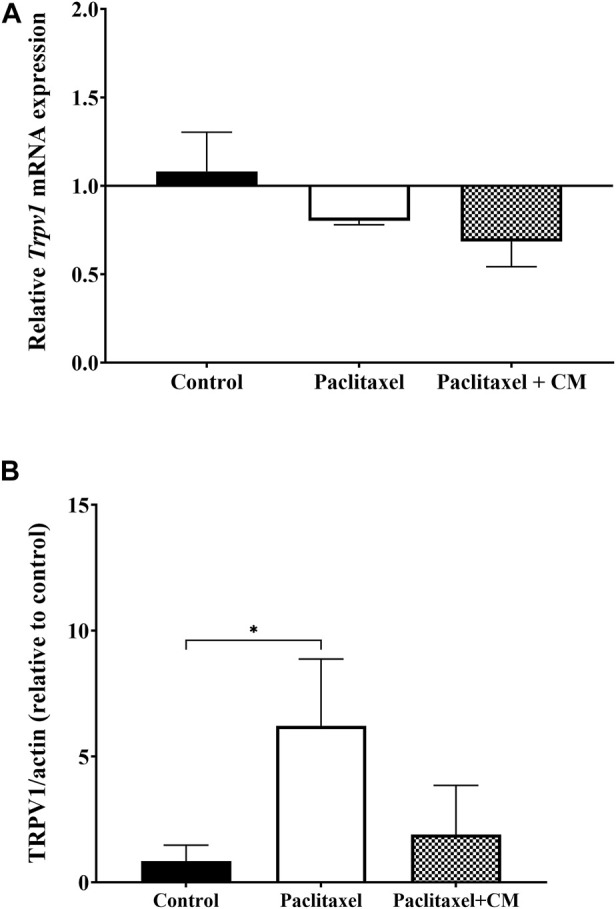
Effect of oral CM extract administration on the relative mRNA and protein expression of TRPV1 in mice treated with paclitaxel. CM extract (250 mg/kg) was administered to female BALB/c mice for 10 days (5 days before paclitaxel injection and 5 days during paclitaxel injection). At day 7 post-paclitaxel injection, the spinal cord was dissected, and the relative expression of Trpv1 mRNA **(A)** and protein expression of TRPV1 **(B)** of the mice were measured. Each bar represents the median and interquartile range of values obtained from 3–4 animals per treatment group. *p < 0.05 compared to the paclitaxel-treated group (Kruskal–Wallis test followed by Dunn’s multiple comparison *post hoc* test).

The mRNA expression of Trpv1 in the spinal cord of animals treated with paclitaxel was not altered compared to those treated with vehicle only (control animals) (*p* > 0.05, Figure 8A). The mRNA expression of Trpv1 in the spinal cord of animals treated with CM was also not altered compared to that in the control or paclitaxel-treated mice (*p* > 0.05, Figure 8A). Unfortunately, the Trpv1 mRNA expression was undetectable in most samples of the brain and paw skin of these mice.

In contrast, the protein expression of TRPV1 in the spinal cord was significantly elevated in the paclitaxel-treated group compared to the control group (*p* < 0.05, Figure 8B). TRPV1 expression was not significantly altered in CM-treated group compared to vehicles-only control group but the CM-treated group showed a trend towards reduction in TRPV1 protein expression when compared to paclitaxel (*p* > 0.05).

### 3.7 Effect of CM extract administration on microglia activation markers

**FIGURE 9:**
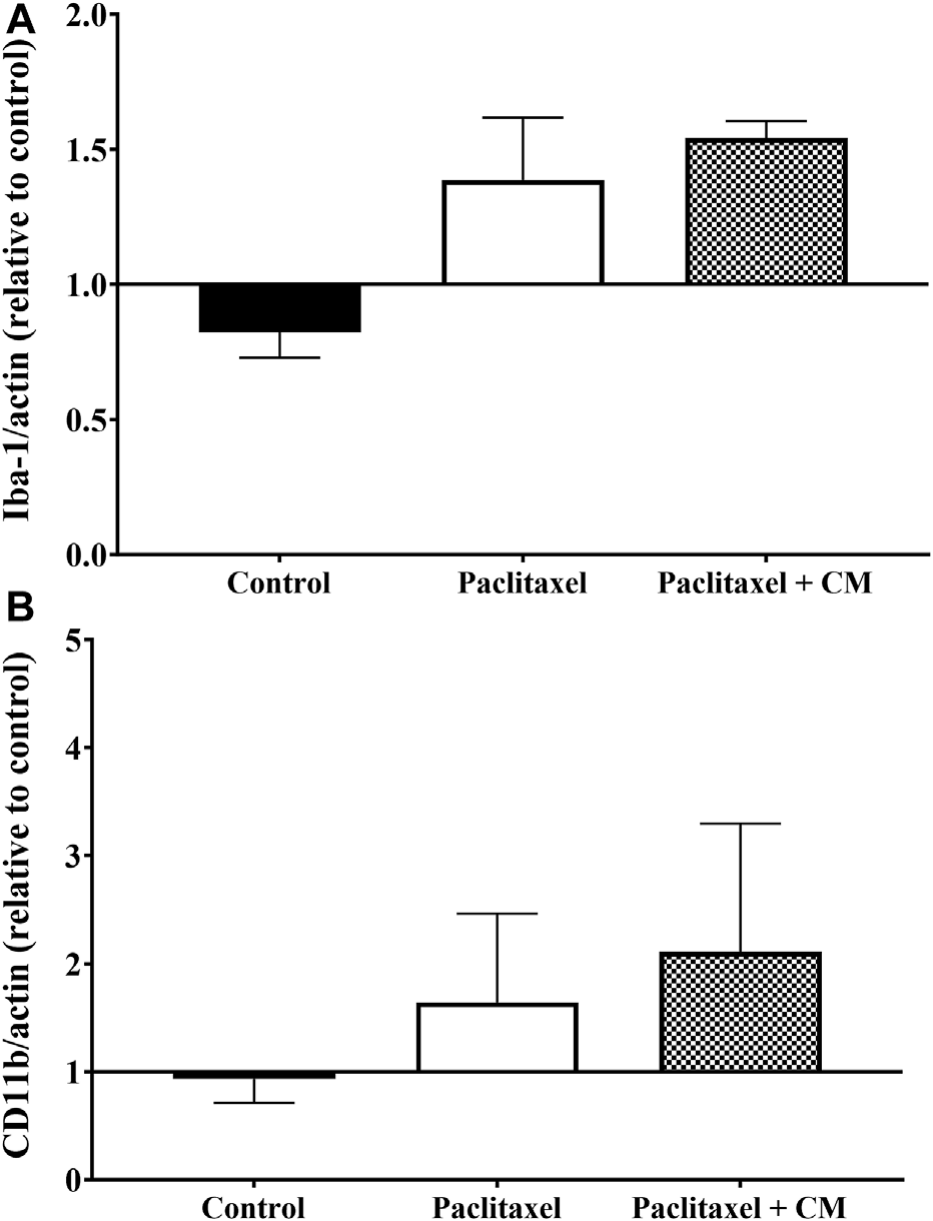
Effect of oral CM extract administration on protein expression of microglia activation markers in mice treated with paclitaxel. CM extract (250 mg/kg) was administered to female BALB/c mice for 10 days (5 days before paclitaxel injection and 5 days during paclitaxel injection). At day 7 post-paclitaxel injection, the spinal cord was dissected, and the protein expression of Iba-1 **(A)** and CD11b **(B)** of the mice was measured. Each bar represents the median and interquartile range of values obtained from 4–8 animals per treatment group.

Administration of paclitaxel alone or paclitaxel +250 mg/kg CM extract did not change the microglia status in the spinal cord of mice as demonstrated by the unchanged levels of Iba-1 and CD11b compared to vehicle-only treated mice (Figure 9).

## 4 Discussion

Herbal medicine and plant extracts have attracted considerable attention as adjunct or alternative therapeutic potentials for the treatment and prevention of neuropathic pain (Ebrahimi et al., 2019; Oveissi et al., 2019; Wu et al., 2019). The use of CM as an analgesic agent has also been the focus of attention in scientific research. Extracts of CM have been reported to have anti-inflammatory and analgesic properties *in vitro* and *in vivo*. CM isolates showed a neuroprotective effect on 1-methyl-4-phenylpyridinium (MPP^+^)-induced neuronal cell death in SH-SY5Y cells *in vitro* (Xu et al., 2011; Xu et al., 2012). Indeed, studies using CM in combination with frankincense (Hu et al., 2017) and with other herbal extracts such as beta-caryophyllene combined with N-palmitoylethanolamide and carnosic acid (available commercially as Noxiall^®^) (Fotio et al., 2019) have shown that chronic administration of these herbal combinations for 14 (Fotio et al., 2019) and 16 (Hu et al., 2017) days alleviated thermal hyperalgesia and mechanical allodynia in chronic constriction injury (CCI)-induced neuropathic pain *in vivo*. However, the individual analgesic effect of CM whether acute or chronic on neuropathic pain has not been evaluated before in any animal model of neuropathic pain. Therefore, in this study, we investigated CM analgesic activities in an animal model of CINP, particularly PINP. We have used treatment and prevention protocols to evaluate the acute and chronic effect of CM on PINP, respectively. Our study showed that CM extract both alleviated painful PINP and ameliorated the development of PINP in female mice, without affecting their locomotor activities, probably through peripheral inhibition of TRPV1 activity, restoration of spinal cord TRPV1 protein expression, and activation of cellular antioxidant defense.

In our study, we assessed neuropathic pain symptoms such as thermal hyperalgesia and mechanical allodynia. Consistent with our previous studies (Parvathy and Masocha, 2012; Masocha, 2014; Masocha and Thomas, 2019; Thomas et al., 2020; Al-Romaiyan and Masocha, 2022), paclitaxel induced thermal hyperalgesia and mechanical allodynia in female mice 7 days post-paclitaxel injection. In our treatment protocol where we treated female mice acutely (for up to 3 h) with CM extract following the establishment of PINP, paclitaxel elicited a marked increase in heat sensitivity which was decreased by CM extract. CM extract reduced thermal hyperalgesia in a dose-dependent manner. The peak antihyperalgesic effect of CM extract was achieved at 1.5 h after CM administration when the CM dose of 250 mg/kg produced a complete restoration of heat sensitivity to vehicle-treated control mice levels. This may suggest that CM could be used to treat painful PINP. In addition, pretreating female mice with CM extract before the development of PINP significantly ameliorated the paclitaxel-induced thermal hyperalgesia and mechanical allodynia, suggesting that CM could have the potential to prevent PINP development. Of note, these effects of CM extract were not attributed to changes in locomotor activities because there was no motor impairment of the CM-treated mice as evident by the unchanged time spent on the rotarod and rotational velocity at which the animals fell off the rotarod apparatus compared to vehicle-treated mice, which is consistent with a previous study (Fotio et al., 2019).

The involvement of TRPV1 channels in the development of neuropathic pain is well known (Boyette-Davis et al., 2015). The activation of TRPV1 is one of the major mediators in pain sensitization. Modulation and blocking of the activity of TRPV1 channels can either be achieved by functional antagonism using pharmacological blockers or through pain desensitization by using a high dose of TRPV1 agonists. Pharmacological antagonists of TRPV1 have been shown previously to attenuate thermal hyperalgesia in a mouse model of PINP (Chen et al., 2011), and thus these antagonists could be important therapeutic candidates in the management of PINP. Our data have shown that paclitaxel elevated the expression of TRPV1 protein in the spinal cord, and CM showed a non-significant downward trend in the protein expression of TRPV1 in the mice with PINP. Therefore, the involvement of TRPV1 channel activity in the antinociceptive action of CM extract was investigated, using a low dose of capsaicin as a selective TRPV1 agonist. Capsaicin selectively stimulates and, at high doses, defunctionalizes TRPV1, resulting in an initial increase and then blockade of pain sensation (Hayman and Kam, 2008; Abdel-Salam and Mózsik, 2023). The capsaicin-induced nociception test has been used since 1992 to investigate the function of TRPV1 in the disease state or modulation of these channels by pharmacological agents (Sakurada et al., 1992). CM extracts significantly reduced capsaicin-induced nociceptive behavior in mice when CM was administered before capsaicin injection into the dorsal surface of the mouse paw as evident by the reduction in the number and duration of the licking, biting, flinching, and paw-lifting responses of mice. These data suggest the involvement of TRPV1 in the antinociceptive effect of CM extract in this animal model.

There is a heightened state of oxidative stress associated with PINP, and the expression of major antioxidative stress markers is altered in the dorsal root ganglia (Zhao et al., 2019), spinal cord (Zhao et al., 2021), and paw skin (Al-Romaiyan and Masocha, 2022) of animals with neuropathic pain. NRF2 is a major transcription factor that protects cells against oxidative stress, and it regulates endogenous antioxidant defense through promoting the expression of a vast range of cytoprotective genes including *SOD2* and *HO-1* (Raghunath et al., 2018; He et al., 2020; Zhou et al., 2021). In the current study, CM extract increased the mRNA expression of Nrf2 but not *Sod2* and *Hmox1* in the paw skin of mice treated with paclitaxel. This suggests that CM may ameliorate PINP through activating cellular antioxidant defense in the peripheral regions involved in pain transmission, and this CM-induced pain alleviation does not involve SOD2 and HO-1, as the expression of these two genes did not change in the paw skin of mice treated with CM compared to paclitaxel-treated mice. No changes in the genetic expression of Nrf2, Sod2, and Hmox1 were observed in the brain or spinal cord. However, we cannot rule out their involvement yet in the CM-induced antioxidant expression. It is worth noting that our experiments measuring the expression of these antioxidants in the CNS were conducted using a complete half of the brain and whole spinal cord, and this could have offset any changes in the genetic expression of these antioxidants in specific brain or spinal cord areas following CM treatment. It will be intriguing to measure the expression of these antioxidants in specific regions involved in central pain transmission.

Numerous studies have demonstrated a critical role of microglia in the development of neuropathic pain (Raghavendra et al., 2003; Tsuda et al., 2003; Coull et al., 2005; Ji and Suter, 2007; Ji et al., 2013). Microglia activation in the spinal cord can be determined by measuring the expression of markers such as Iba-1 and CD11b. Our data showed that paclitaxel did not change the protein expression of these two markers in the spinal cord of mice, and CM also had no effect on their expression. This may suggest that CM alleviated PINP independent of microglia activity.

In conclusion, this study shows that CM extract has antinociceptive activity in an animal model of PINP. Our data show that acute CM treatment alleviated paclitaxel-induced thermal hyperalgesia, indicating that CM could be used to treat established PINP. Chronic treatment with CM before the development of PINP also ameliorated paclitaxel-induced thermal hyperalgesia and mechanical allodynia, demonstrating that CM may prevent the development of PINP. The possible underlying mechanism to the analgesic activity of CM may involve TRPV1 inhibition and cellular antioxidant activation, namely, NRF2 activation in the periphery. Our findings suggest that CM could be used as a therapeutic alternative in the management of PINP.

## Data Availability

The raw data supporting the conclusion of this article will be made available by the authors, without undue reservation.
